# A Straightforward HPV16 Lineage Classification Based on Machine Learning

**DOI:** 10.3389/frai.2022.851841

**Published:** 2022-06-23

**Authors:** Laura Asensio-Puig, Laia Alemany, Miquel Angel Pavón

**Affiliations:** ^1^Cancer Epidemiology Research Programme, Catalan Institute of Oncology, Bellvitge Biomedical Research Institute (IDIBELL), L'Hospitalet de Llobregat, Barcelona, Spain; ^2^Centro de Investigación Biomédica en Red de Epidemiología y Salud Pública (CIBERESP), Madrid, Spain

**Keywords:** Human Papillomavirus (HPV), cancer, prognostic and predictive factors, classification, machine learning, HPV16 lineage

## Abstract

Human Papillomavirus (HPV) is the causal agent of 5% of cancers worldwide and the main cause of cervical cancer and it is also associated with a significant percentage of oropharyngeal and anogenital cancers. More than 60% of cervical cancers are caused by HPV16 genotype, which has been classified into lineages (A, B, C, and D). Lineages are related to the progression of cervical cancer and the current method to assess lineages is by building a Maximum Likelihood Tree (MLT); which is slow, it cannot assess poor sequenced samples, and annotation is done manually. In this study, we have developed a new model to assess HPV16 lineage using machine learning tools. A total of 645 HPV16 genomes were analyzed using Genome-Wide Association Study (GWAS), which identified 56 lineage-specific Single Nucleotide Polymorphisms (SNPs). From the SNPs found, training-test models were constructed using different algorithms such as Random Forest (RF), Support Vector Machine (SVM), and K-nearest neighbor (KNN). A distinct set of HPV16 sequences (*n* = 1,028), whose lineage was previously determined by MLT, was used for validation. The RF-based model allowed a precise assignment of HPV16 lineage, showing an accuracy of 99.5% in the known lineage samples. Moreover, the RF model could assess lineage to 273 samples that MLT could not determine. In terms of computer consuming time, the RF-based model was almost 40 times faster than MLT. Having a fast and efficient method for assigning HPV16 lineages, could facilitate the implementation of lineage classification as a triage or prognostic marker in the clinical setting.

## Introduction

A total of 5% of worldwide cancers are caused by the Human Papillomavirus (HPV) being cervical cancer the fourth most common cancer in women (Arbyn et al., 2020). Although the incidence of cervical cancer has decreased over the last years (Arbyn et al., 2011; Van Dyne et al., 2018) due to the implementation of screening methods (Brisson et al., 2020) and it may decrease in the following years due to vaccination (Bruni et al., 2021; Falcaro et al., 2021), an estimated 570,000 women were diagnosed with cervical cancer worldwide in 2018 (Bray et al., 2018). Moreover, the incidence of non-cervical cancers has increased in recent years. While in cervical cancer HPV prevalence is close to 100%, in other HPV-associated anogenital cancers viral prevalence rates differ according to the anatomical site: anus (88%; Alemany et al., 2015), vagina (74%; Alemany et al., 2014), penis (33%; Alemany et al., 2016), vulva (29%; de Sanjosé et al., 2013), and oropharynx (29–70%; Stein et al., 2015).

HPV high-risk types (HR-HPV) include predominantly, alpha 9 (HPV 16/31/33/35/52/58), alpha 7 (HPV 18/39/45/59/68), alpha 6 (HPV 56/66), and alpha 5 (HPV 51) genus, but HPV16 is by far the most common HR-HPV type, which contributes to 70–75% of all cervical cancers and is found in 40–60% of cervical intraepithelial neoplasia 2 (CIN2+; Bzhalava et al., 2013). However, only 5% of persistent HPV16 infections will evolve to high-grade lesions, and from those, a small proportion will progress to invasive cancer. Although it remains unclear why some HPV16 infections progress while others are cleared spontaneously, viral genome variability has been described as a key factor that could play a crucial role in the progression toward high-grade lesion or invasive cancer risk (Cullen et al., 2015). HPV16 was classified accordingly to viral genome variability in different lineages (A, B, C, and D) and sublineages (A1-4, B1-3, C1, D1-3) by Burk et al. (2013). HPV16-A lineage is the most prevalent type worldwide, while HPV16-D is the most aggressive type associated with cervical cancer risk (Gheit et al., 2011; Mirabello et al., 2016; Clifford et al., 2019).

In the 90's, the HPV genotype and HPV16 variants were determined according to the L1—Open Reading Frame (ORF) region that was amplified and sequenced (Ho et al., 1991; Chen et al., 2005). The implementation of New Generation Sequencing (NGS) techniques allowed us to perform bulk experiments and obtain longer sequences beyond the L1 ORF. Full viral genome sequencing resulted in the discovery of more lineages and genome variants (Burk et al., 2013). High-throughput sequencing as Illumina or Ion Torrent (Cullen et al., 2015) methods leads us to read the full viral genome. Before estimating the similarity between genomes, sequence samples are aligned to the reference HPV16 sequence (NCBI genome IDs: NC_001526.4). Then, a Maximum Likelihood Tree (MLT) is built altogether with a set of known-lineage HPV genomes used as a reference to assign specific lineages (Smith et al., 2011). New samples are placed on the phylogenetic tree according to their similarity with the reference sequences. Finally, the researcher manually assigns a lineage for the sample of interest, looking at where the sample has been located on the phylogenetic tree.

However, since the current method uses the entire genome sequence, poor coverage samples and samples showing gaps or missing fragments are difficult to classify. Building a phylogenetic tree is a time-consuming method when the sample size is big, which may take a long time to process depending on the computer used and finally, the lineage assignment is done manually. As MLT classification is directly influenced by the operator's expertise, reproducibility and standardization of the method may vary. To improve the HPV16 lineage assessment, we propose a new model that uses a few positions on the HPV16 genome to assess lineage and it does not require visual control, which makes the process faster and reproducible.

In this study, we describe a new code that can be used to efficiently assign HPV16 lineages. Using a Genome-Wide Association Study (GWAS), we tested all the positions of the HPV16 genome that are known to be unique to a single lineage or sublineage. Then, using machine learning algorithms, we trained and tested different models using reference and known samples for these positions. The code has been developed with the R language and it has been validated with more than 4,000 HPV16 genomes. Having a fast and efficient method for assigning HPV linages will help clinics to provide better-informed prognoses and help to define screening and treatment decision strategies.

## Materials and Methods

### Samples

HPV16 genome sequences were used to find the lineage-specific SNPs and to build the model to assess lineage. Reference samples were obtained from two different sets of known-lineage HPV16 genomes: one set was described by Burk (*n* = 46; Smith et al., 2011) and the other was obtained from the Papillomavirus genome database (PAVE) webpage (*n* = 10; Supplementary Material 1). To define the lineage-specific positions for HPV16A, HPV16B, HPV16C, and HPV16D and to build the training-test models we used the reference samples and all the complete HPV16 genomes from NCBI (*n* = 588), downloaded from NCBI nucleotide dataset by keyword search “txid333760 complete genome;” Species: Viruses; Molecular types: Genomic DNA/RNA; Sequence type: Nucleotide accessed on July 30, 2021.

Validation of the model was performed with two different sets of samples, the first set of 1,028 HPV16 samples collected and sequenced in our laboratory, and the second set of 3,898 samples (which included the complete genomes and other almost complete genomes) were downloaded from NCBI nucleotide dataset by keyword search txid333760; Species: Viruses; Molecular types: Genomic DNA/RNA; Sequence type: Nucleotide; Release Date: From 0000/01/01 to 2022/03/24; Sequence length: from 7,000 to 8,500; accessed on March 24, 2022.

All samples were aligned on the HPV16 reference genome (GenBank Accession code: K02718.1) with MAFFT (v7.475) software using “–add” and “–keeplength” options (Katoh et al., 2019). The HPV16 reference genome, which is the HPV16-A1 sublineage has been added to the reference sample set (*n* = 57).

### Lineage Assessment

The HPV16 lineage was assigned to the 588 NCBI samples using the current lineage assignment process described by Burk (Burk et al., 2013; Cullen et al., 2015) based on phylogenetics, which we will henceforth call Maximum Likelihood Tree (MLT), as it is based on this the Maximum Likelihood algorithm. The process consists of building a phylogenetic tree with altogether known lineage sequences and samples of interest. Phylogenetic analysis was conducted using MEGAX (Tamura et al., 2021) (v10.2.4) using the 57 reference samples plus the 588 NCBI previously aligned samples. To build the phylogenetic tree, we first calculated the genomic variation in a group of sequences with the Maximum Likelihood statistical method applying the Tamura-Nei correction model for nucleotide substitution. The process was replicated 100 times with the bootstrapping method. Finally, a tree was built, and lineage was assigned to each sample accordingly to the closest reference sample and results were manually annotated. Not all samples were assigned to a lineage, since some sequences were not placed in the main lineage branches, so they were classified as “n” or unknown lineage.

### Detection of Main Nucleotides Related to Lineage

A Genomic Wide Association Study (GWAS; Manolio, 2010) was performed on the reference and NCBI sequences (*n* = 645) to find differences between lineages within the HPV16 genome. The 7,906 base pairs that make up the viral genome have been traced to detect mutations. Known positions with two or more alleles with a minimum variant frequency (MVF) of 0.05 and a call rate higher than 95% were called SNP candidates. A generalized linear model (GLM) with a binomial distribution and a logit function was used to test the relationship between each SNP candidate and the HPV16 lineage. *P*-values were adjusted by False Discovery Rate (FDR) and only SNPs with a *p*-value lower than 0.05 were considered significant.

### Training a New Model to Assess Lineage

To assess lineage with the SNPs described in the previous step we opt for training-test models. Different algorithms had been used to train models: Random Forest (RF), Support Vector Machine (SVM) and K-nearest neighbor (KNN), and Classification and Regression Trees (CART). The model was built with a total of 646 samples, including the 588 NCBI complete HPV16 genomes, the 57 reference samples, and a new sample called the “n-sample.” The n-sample had no information and was composed of 7,906 unknown nucleotides (“n”), to assign unknown lineage to those samples with poor coverage. The 80% (*n* = 518) of the samples had been used for training and testing the model, while the remaining 20% (*n* = 128) had been used for the validation. For a better estimation, samples have been randomly mixed 100 times creating different training and test groups with the k-fold cross-validation method, and the model has been trained and tested for each new dataset. Accuracy, Kappa constant, and the testing confusion matrixes have been used to compare models and to choose the best model for lineage assessment.

### Validate the New Model

Finally, validation has been performed to test the new model with two datasets of samples. The model has been validated with 3,898 genomes downloaded from NCBI which included both complete genomes and almost complete genomes and with a dataset composed of 1,028 HPV16-positive samples, that were selected from the archive of HPV tumors collected for the RIS HPV TT, VVAP, and Head and Neck studies (De Sanjose et al., 2010) and coordinated by the Catalan Institute of Oncology (ICO). Formalin-fixed paraffin-embedded (FFPE) specimens were sequenced with the HPV16 assay designed for the Ion Torrent Sequencing platform, which covers more than 80% of the viral genome (Cullen et al., 2015). Therefore, this last step of validation has tried out the model with a set of incomplete genomes as the sequencing assay was designed to amplify low-quality archival DNA.

In both datasets, lineage was first assessed with MLT, to then compare the quality of the new lineage assessment done by the machine learning model. Both GWAS and the training-test model has been performed using R language under 3.6.3 version and the code is available on www.github/INCALAB-PREC/HPV16-linpred/.

## Results

**Figure 1:**
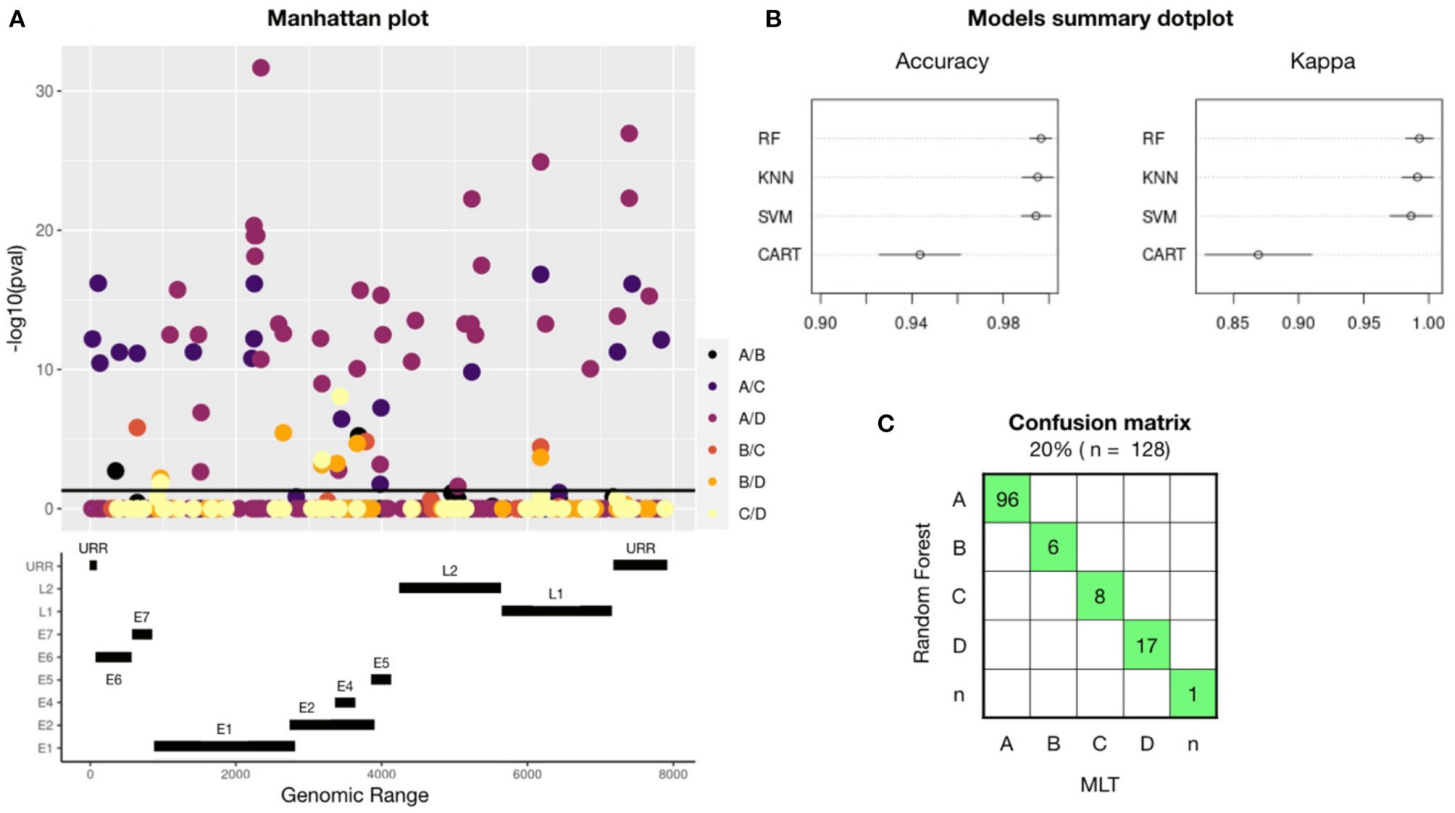
A total of 56 lineage-definers SNPs were found in the GWAS, which were used to build four machine learning models, being RF the best model to predict HPV16 lineage. **(A)** Manhattan plot showing all the nucleotides in the HPV16 genome with any mutation (MAF > 0.05 and CR < 95%). SNP candidates have been colored differently according to which lineage they are specific for. Therefore, one SNP candidate can define two different lineages and it will be plotted twice. SNPs significantly related (*p* < 0.05) with any lineage are plotted above the black line. The genomic map under the Manhattan plot shows the positions of the HPV16 genes. **(B)** Accuracy and Kappa of the different training-test algorithms build to predict HPV16 lineage. Models used are Random Forest (RF), Support Vector Machine (SVM), K-nearest neighbor (KNN), and Command Assessment of Readiness and Training (CART). Kappa is a metric that compares an observed Accuracy with an expected Accuracy obtained by cross-validation (100-fold). When both, accuracy and kappa are 1, the model is perfect. **(C)** Confusion matrix for the Random Forest-based model using the validation set of samples. In green, samples that had been assessed equally with both methods: RF and Maximum Likelihood Tree (MLT).

A GWAS performed on 645 HPV16-reference genomes showed 56 SNPs that are unique for one or more HPV16-lineages: A, B, C, or D (Figure 1A). Significant SNPs were spread out into the full genome. Gene E1 had a total of 16 lineage-definers SNPs, followed by E2 (10 SNPs), L2 and URR (7 SNPs), E6 (4 SNPs), E5 and L1 (3 SNPs), E7 (1 SNP), and 5 SNPs were found in a non-codifying region. Most of the differences in nucleotides were found between A and D or C lineages.

The training-test models were built using the 80% (*n* = 518) of the HPV16 dataset randomly selected and considering only the 56 lineage-specific positions found in the GWAS. The 100 k-fold cross-validation method has been applied and the dataset has been resampled 100 times in train and test groups. Each new dataset group was trained and tested to improve the estimated values of the model. Figure 1B shows a comparison between the models used, revealing that the best model to assess HPV16 lineage was the Random Forest (RF) algorithm, with an accuracy of 0.99 (CI:95%), followed by Support Vector Machine (SVM) and K-nearest neighbor (KNN); with a mean accuracy of 0.98 (CI: 95%) for both. Validation of the models was performed with the remaining 20% of the dataset (*n* = 128). To build the confusion matrix, lineage was assessed using the three models (RF, SVM, and KNN) and individually compared with the lineage assessed by MLT. Random Forest was the model with less error since all the assessed lineages match with MLT and were selected for the next validation steps (Figure 1C). Despite the high accuracy of SVM and KNN models, both failed in one single sample.

**Figure 2:**
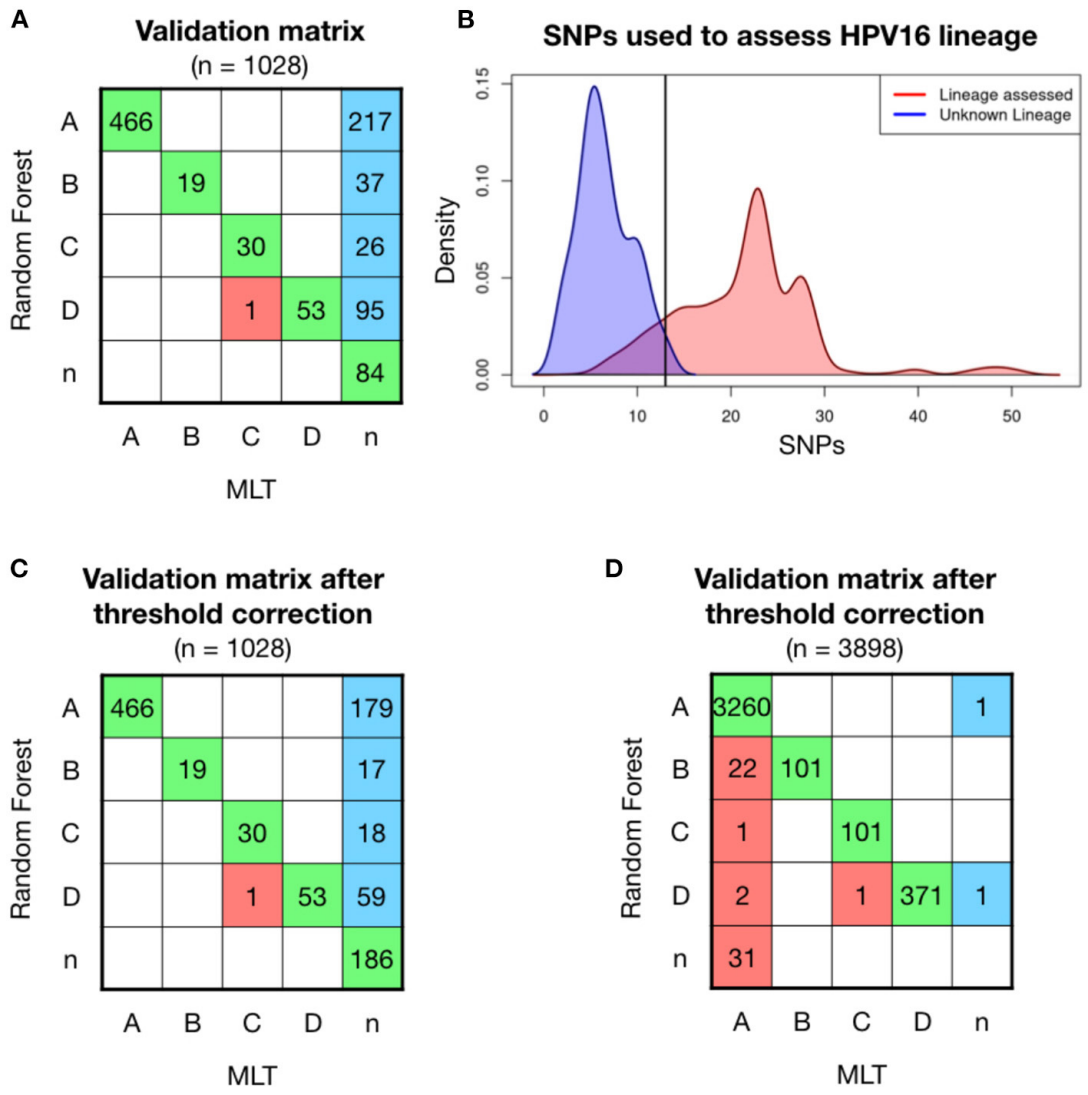
Model validation on 1,028 patient' HPV16 sequences showed higher ratios of classification with Random Forest (RF) model than with Maximum Likelihood Tree (MLT). **(A)** RF validation was performed on 1,028 samples and shown in a confusion matrix. Samples receiving the same classification from both pipelines are colored in green, while samples that are not classified with the same lineage are shown in red. In the last column, sequences that RF-based model could assign for the first time and MLT could not. **(B)** Density curves of the number of covered lineage specific SNPs for each sample in function if their lineage has been assessed by RF (red) or not (blue), shows that the smaller number of known SNPs makes more difficult for the RF model to assess lineage. The black line corresponds at the intersection point between the two densities curves, where we had defined a threshold, where samples with <13 SNPs will be considered as the unclassified lineage or “n.” **(C)** Validation matrix after threshold correction, discarding all the samples that have less than 13 known lineage-definers SNPs. Notice that the threshold only changes the blue column, increasing the n-samples from 87 to 182. **(D)** Validation matrix using 3,898 HPV16 genomes available in the nucleotide database from NCBI. Samples with <24 lineage-dependent SNPs had been classified as n-sample.

Further validations were carried out with two independent set of samples, the first one included 1,028 HPV16 positive samples, whose genome was partially obtained from FFPE archive samples. Most of the high coverage samples were classified with the same lineage as the MLT method did, shown in green in the confusion matrix (Figure 2A). Only one sample was differently classified between models (in red). MLT lineage classification is a challenge in low coverage samples, since out of 1,028 samples only 569 (56.1%) could be evaluated. In contrast, RF model has been able to assess lineage in 943 (93.0%) of these sequences. Therefore, if the MLT model is considered as the reference method for assessing HPV16 lineage, the RF model has an error of 0.17%. A total of 375 samples with average coverage have been assessed for the first time (in blue). However, we have no way of confirming that these samples have been properly classified. Lineage could not be assessed in 84 samples by either method, which has been classified as “n” samples. The coverage of most of these samples is poor, although some samples with good coverage were found in the unclassified group.

To understand in which conditions the RF model can assign lineage, different statistical analyses had been performed. Lineage has been assessed with a median of 24 known SNPs out of the 56 lineage-specific SNPs in a single sample (in red), while the 84 sequences that no lineage could be assigned had <15 known SNPs (in blue; Figure 2B). Therefore, it must exist a minimum number of SNPs to successfully run the model. We have fixed a threshold at the intersection of the two density lines, which is 13 SNPs, and sequences with <13 out of the 56 lineage-definer SNPs will be directly assigned as unclassified lineage - “n.” Applying the threshold, the confusion matrix slightly changes, losing a total of 102 samples that will be considered as “n” instead of the predicted lineage (Figure 2C). None of the samples equally assigned for both methods, RF and MLT, has been affected by the application of the threshold. After the correction, the percentage of lineage assignment decreased from 93.0 to 82.9%. Discarded samples included sequences of both good and bad coverage samples.

RF model has been validated with a second set that includes all the HPV16 genomes available in the NCBI dataset in March 2022. Lineage has been previously assessed by the MLT method and then has been assessed with the random-forest algorithm. The accuracy of the validation matrix is 98.9% (*p* < 0.001) and the error when assigning the lineage is <1.5%. However, a set of samples classified with the MLT method as HPV16-A lineage had been classified as B (*n* = 28) and D (*n* = 12) using the RF model. Discarding those who had <24 known SNPs the matrix improves, which indicates that the loss of certain SNPs after sequencing incomplete genomes, could influence the classification model accuracy. However, 22 samples are still classified as B instead of A (Figure 2D). This is probably due to a large number of HPV16-A samples included in the validation step compared to the other lineages. Although the error in lineage A classification is only 0.67%, most of the errors accumulate in B, which is the closest lineage to A, and overall, one of the less frequent lineages. In turn, all samples initially classified using MLT as B were well-classified as B using the RF model, which confirms that the model works to classify lineage B.

As the prevalence of HPV16-A lineage is higher in the world, for this reason, all the possible HPV16 datasets will have an important bias. We evaluate the model with a balanced dataset for each lineage. Sets of 200 lineage-balanced samples had been created randomly selecting 50 samples of each lineage from the full NCBI dataset (*n* = 3,898). The validation of the model, repeated with 10 different random sets shows an accuracy of 0.986 (95% CI: 0.958–0.997). The A-samples misclassification to B almost disappears (Supplementary Material 2).

In both pipelines, samples must be aligned to a reference genome. MAFFT takes an average of 2 min to align a total of 100 HPV16 genomes. It takes ~40 min to calculate the distances between samples with the MLT algorithm and to build a phylogenetic tree (bootstrapping samples 100 times) (Table 1). Followed by the annotation step, where the operator annotates manually the lineage by looking at the phylogenetic tree, which may take between 30 and 40 min depending on the skills of the worker. Using the developed code in this project, it only takes 0.97 s (SD = 0.43, repeated 25 times) to load the samples in Fasta format, assess lineage with the RF model and annotate lineage. For 100 samples, the new RF pipeline is almost 40 times faster than the current MLT pipeline. By increasing the number of samples to be tested, the difference between models becomes much larger. To assign lineage in our 1,028 HPV16 genomes dataset, the RF model was almost 40,000 times faster than MLT, since the process to build the MLT and annotating lineage lasted approximately up to 30 h, while the RF model took only 2,81 s (SD = 0.15, repeated 10 times).

**Table 1:** HPV16 lineage classification is faster with the Random Forest pipeline.

**100 HPV16 samples**	**Current pipeline (MLT)**		**New pipeline (RF)**	
	**Software/**	**Time**	**Software/**	**Time**
	**method**	**(min)**	**method**	**(min)**
Alignment	MAFFT	2	MAFFT	2
Algorithm	MEGAX/MLT	40	R/RF	0.97 s
Annotation	Manually*	30–40*	R	

*The time for both pipelines was calculated on a set of 100 HPV16 sequences and tracked in a computer with the following features: UBUNTU 20.04 with 4 GHz Intel Core i7 and 16 GB of RAM*.

For both pipelines, samples were aligned on the reference HPV16 genome with MAFFT software using “—keeplength” function. For the current pipeline we used MEGAX software to calculate the distance between sequences with the Maximum Likelihood tree (MLT) method and to build a phylogenetic tree. The new pipeline has been developed with R language and uses the Random Forest (RF) algorithm from the “caret” library. While the R code generates an output with the samples ID and the assigned lineage, the current pipeline requires manual annotation and the estimated time^*^ may depend on the operator's skills.

### Sublineage A

From the reference genome set (*n* = 645), a total of 481 HPV16-A samples had been selected, all of them assessed with A-lineage by both models, MLT and RF. Nucleotide differences between 0.5 and 1% of the complete genomes are used to define the sublineages (Burk et al., 2013), and HPV16-A lineage is classified in A1, A2, A3, and A4 groups. As HPV16-A1, A2, and A3 sublineages are more similar to each other and have a similar contribution on HPV-associated cancers than A4, we decided to cluster them into a single group called A123. A total of 67 positions were classified as SNP candidates (CR > 95% and MAF > 0.05), but the GWAS only assessed 17 significantly SNPs associated with A123 or A4 sublineage.

**Figure 3:**
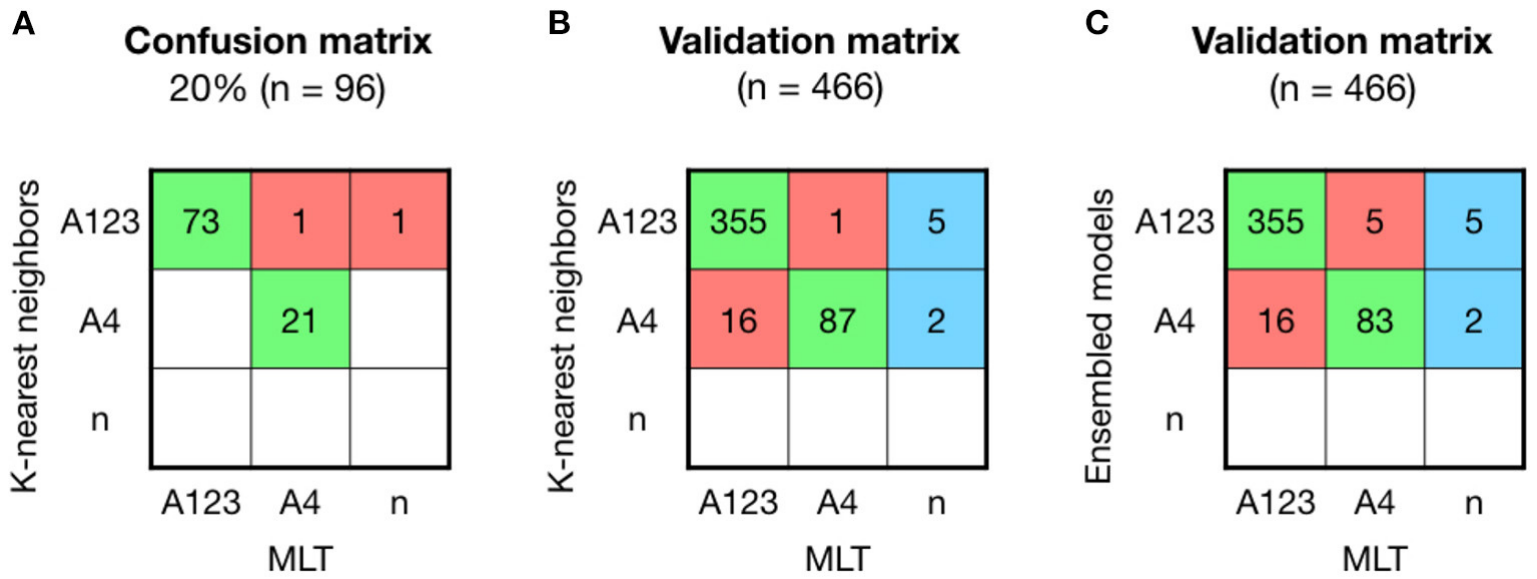
Comparison between KNN, MLT and ensembled models to assign sublineage A shows good results but with higher error than the lineage model. **(A)** K-nearest neighbors (KNN) confusion matrix on the 20% of the HPV16-A reference sequences that were not used to build the model. **(B)** KNN validation was performed on 466 HPV16 patient's sequences and shown in a confusion matrix. Samples receiving the same classification from both pipelines are colored in green, while samples that are not classified with the same sublineage are shown in red. In the last column, sequences that KNN-based model could assign for the first time and Maximum Likelihood Tree (MLT) could not. **(C)** Ensembled model by majority vote was validated on the 466 HPV16-A patient's genomes.

An 80% of the samples were used to build the models, and from the five machine-learning models used in this study, RF and KNN were the models with better results to predict sublineage A. KNN model obtained an accuracy of 0.979 (95% CI: 0.926–0.997), which showed similar values than RF with an accuracy of 0.968 (CI: 0.911–0.993). After resampling and building the model 100 times, models were validated other 20% of the samples (*n* = 96). The validation matrix showed two mismatches between KNN and MLT (Figure 3A), instead of the three mismatches produced by the RF model, even showing the same accuracy values. A second validation was performed with the patient's sequenced HPV16-A samples (*n* = 466) obtained from the project led by ICO (Figure 3B). The accuracy of predicting sublineage A123 or A4 was 0.939 (95% CI: 0.914–0.959), being lower than the lineage model accuracy.

The training-tests with an accuracy higher than 95% (RF, KNN, and SVM) were ensembled by the majority vote method. The ensemble model did not improved the KNN prediction (Figure 3C).

## Discussion

The HPV16 lineage classification needs to be more efficient if we ever want to implement it as triage or prognostic marker in the clinical setting. Here we describe a faster and automated new model based on machine learning that efficiently classifies HPV16 sequences into lineages and requires lower sequence coverage if compared with the current method.

The current classification model calculates the similarity between samples and reference HPV16 genomes using the Maximum Likelihood estimation to classify the sampled sequence into a given lineage. To work, the MLT algorithm requires, as input, the whole HPV16 genome (7,906 base pairs), therefore, sampled sequences with large uncovered regions cannot be assigned to any lineage. We performed a genomic wide association study in which we identified 56 SNPs that are HPV16-lineage specific. The subset of SNPs included in the RF model is mainly lineage definers, our results are in agreement with previously described studies using phylogenetic reconstruction and classification to assign HPV16 variants to clinical sample (Ou et al., 2021).

Working with 56 SNPs instead of the full genome sequence, we can develop more efficient and faster models than the current model used for HPV16 lineage classification. Among different training-test models used to assess lineage based on the 56 SNPs, Random Forest was the best one, with an accuracy close to 100%. Using the RF to classify more than 1,000 samples we could assign a lineage to 93% of the samples, whereas using MLT we assigned a lineage to 56.1%. If the MLT model is considered as the reference method for HPV16 lineage classification since it does not exist another method, the new RF-based model would have an error between 0.17 and 1.4% according to both validation matrices. Therefore, from the 273 samples of first-time lineage assessed by RF in the 1,028 patient samples, we may assume that the error is similar, so there would be between 1 or 4 misclassified samples in this group.

Not all the SNPs are equally related to the lineage. A total of 20 out of 56 SNPs used in the model show higher Odds Ratio (OR) values when the relation between nucleotide and lineage is tested, thus lineage assessment could also work with a smaller set of SNPs in each sample. The density histogram showed that at least 13 SNPs must be known to assess lineage with the RF model, in consequence, samples with <13 known SNPs will be considered non-classified samples to avoid errors in low coverage samples. Besides the reduction of data required, if compared to the MLT pipeline, the RF model also allows a much faster process that does not require manual annotation. The RF model is 40 times faster than the MLT model.

Sequencing is becoming affordable to most laboratories, and consequently becoming a part of the clinical setting; however, it generates large amounts of data that may be difficult to analyze, besides being time-consuming. The new model we present here allows a straightforward assignment of HPV16 sequence alignment of virtually all sampled sequences.

The main limitation of this study is that we did not test our model for all sublineages, the training-test models could be only applied for A sublineage. Further studies should investigate the mismatched samples in order to unveil any potential limitation of the RF model for assigning HPV16 lineages. Our model can be implemented to classify HPV genotypes and other HPV lineages. Thus, samples from cervical and anogenital sites that are positive for any HPV type could be assigned to a specific lineage.

Having a fast and efficient method for assigning HPV linages may allow better-informed prognosis and may better guide doctors on the best course for women showing an HPV16 positive test or individuals with HPV positive pre-neoplastic lesions and high-grade lesions. Most of the current screening algorithms, using HPV as a primary test, define that HPV16 positive women should be referred directly to colposcopy, while more than 95% of these infections will be cleared spontaneously during the next 12 months. The identification of HPV16-positive women with a high risk of progression is a key point to develop new diagnostic tools for improving screening or diagnostic specificity avoiding unnecessary methods.

In addition, the computational model described in this work would be easily implementable in a user-friendly software or web interface, which will make easier the introduction of HPV16 lineage classification in the clinical setting.

## Data Availability Statement

The original contributions presented in the study are included in the article/Supplementary Material, further inquiries can be directed to the corresponding authors.

## Ethics Statement

The study was approved by the Ethics Committee of Hospital Universitari de Bellvitge. Written informed consent for participation was not required for this study in accordance with the national legislation and the institutional requirements.

## Author Contributions

LA-P and MP conceived of the presented idea. LA-P developed the theory and performed the computations. LA contributed to the conceptualization of the work. All authors discussed the results and contributed to the final manuscript.

## Funding

This work was supported by a grant from the Instituto de Salud Carlos III (Spanish Government) through the projects PI17/00123 (Co-funded by European Regional Development Fund. ERDF, a way to build Europe) and CIBERESP CB06/02/0073, and the Secretariat for Universities and Research of the Department of Business and Knowledge of the Government of Catalonia grants to support the activities of research groups 2017SGR1085. We thank the CERCA Programme/Generalitat de Catalunya for institutional support. None of these entities played a role in data collection, data analysis, data interpretation, or report writing. All authors had full access to all data in the study and had final responsibility for the decision to submit for publication.

## Conflict of Interest

The authors declare that the research was conducted in the absence of any commercial or financial relationships that could be construed as a potential conflict of interest.

## Publisher's Note

All claims expressed in this article are solely those of the authors and do not necessarily represent those of their affiliated organizations, or those of the publisher, the editors and the reviewers. Any product that may be evaluated in this article, or claim that may be made by its manufacturer, is not guaranteed or endorsed by the publisher.
