# Supplementary material for: A Straightforward HPV16 Lineage Classification Based on Machine Learning
Source: Front Artif Intell. 2022 Jun 23;5:851841. doi: 10.3389/frai.2022.851841 (PMC9260188; doi:10.3389/frai.2022.851841)

**A straightforward HPV16 lineage classification based on machine learning**

**Laura Asensio-Puig^1^, Laia Alemany^1,2^ and Miquel Angel Pavón^1,2^**

*1. Cancer Epidemiology Research Programme, Catalan Institute of Oncology, Bellvitge Biomedical Research Institute (IDIBELL), L’Hospitalet de Llobregat, Barcelona, Spain*

*2. Centro de Investigación Biomédica en Red de Epidemiología y Salud Pública (CIBERESP CB06/02/0073), Madrid, Spain*

**SUPPLEMENTARY DATA**

**Supplementary data 1:** GenBank IDs of the samples used to determine lineage-specific SNPs with GWAS.

A) HPV16 reference samples obtained from the variants section in the PAVE web page.

| Sublineage | GenBank ID | Previously used name |
| --- | --- | --- |
| A1 | [K02718](http://www.ncbi.nlm.nih.gov/nuccore/K02718?report=genbank) | European (E) |
| A2 | [AF536179](http://www.ncbi.nlm.nih.gov/nuccore/AF536179?report=genbank) | European (E) |
| A3 | [HQ644236](http://www.ncbi.nlm.nih.gov/nuccore/HQ644236?report=genbank) | European (E) |
| A4 | [AF534061](http://www.ncbi.nlm.nih.gov/nuccore/AF534061?report=genbank) | Asian; E(As) |
| B1 | [AF536180](http://www.ncbi.nlm.nih.gov/nuccore/AF536180?report=genbank) | African-1; Afr1a |
| B2 | [HQ644298](http://www.ncbi.nlm.nih.gov/nuccore/HQ644298?report=genbank) | African-1; Afr1b |
| C1 | [AF472509](http://www.ncbi.nlm.nih.gov/nuccore/AF472509?report=genbank) | African-2;Afr2a |
| D1 | [HQ644257](http://www.ncbi.nlm.nih.gov/nuccore/HQ644257?report=genbank) | North American (NA)1 |
| D2 | [AY686579](http://www.ncbi.nlm.nih.gov/nuccore/AY686579?report=genbank) | Asian_American (AA)2 |
| D3 | [AF402678](http://www.ncbi.nlm.nih.gov/nuccore/AF402678?report=genbank) | Asian_American (AA)1 |

B) HPV16 reference samples described by Smith et. al. in the article “Sequence imputation of HPV16 genomes for genetic association studies, PLoS One” that can be downloaded in the NCBI genomic database using their corresponding GeneBank ID.

| Sublineage | GeneBank ID | Isolate ID | Previously used name |
| --- | --- | --- | --- |
| A1 | HQ644268 | Qv11687 | E (Prototype 1) |
| A1 | AY686583 | Qv18158 | E (Prototype 1) |
| A2 | AF536179 | w0122 | E (Prototype 2) |
| A3 | HQ644236 | AS411 | E (Prototype 1) |
| A4 | HQ644234 | AS097 | E (Asian) |
| A4 | HQ644248 | IN151168 | E (Asian) |
| A4 | HQ644251 | INJP0168 | E (Asian) |
| A4 | AF534061 | W0724 | E (Asian) |
| A4 | HQ644261 | QV02706 | E (Asian) |
| A4 | HQ644235 | AS310 | E (Asian) |
| B1 | HQ644240 | BF325 | Af1 |
| B1 | HQ644238 | BF215 | Af1 |
| B1 | AF472508 | R872 | Af1 |
| B1 | AF536180 | w0236 | Af1 |
| B1 | HQ644299 | Z122 | Af1 |
| B1 | HQ644290 | Rw768 | Af1 |
| B1 | HQ644296 | Z16 | Af1 |
| B1 | HQ644293 | Rw918 | Af1 |
| B2 | HQ644298 | Z109 | Af1 |
| C0 | AF472509 | R460 | Af2 |
| C0 | HQ644250 | IN272098 | Af2 |
| C0 | HQ644292 | Rw862 | Af2 |
| C0 | HQ644291 | Rw851 | Af2 |
| C0 | HQ644249 | IN221688 | Af2 |
| C0 | HQ644239 | BF236 | Af2 |
| C0 | HQ644237 | BF039 | Af2 |
| D1 | HQ644257 | Qv00512 | NA1 |
| D2 | AY686579 | Qv15321 | AA2 |
| D2 | HQ644270 | Qv13791 | AA2 |
| D2 | AY686582 | Qv15351 | AA2 |
| D2 | HQ644277 | Qv22478 | AA2 |
| D2 | HQ644273 | Qv19067 | AA2 |
| D2 | HQ644281 | Qv24898 | AA2 |
| D2 | HQ644279 | Qv23890 | AA2 |
| D2 | HQ644254 | Qv00124 | AA2 |
| D3 | AF402678 | Qv00995 | AA1 |
| D3 | HQ644247 | IN000078 | AA1 |
| D3 | HQ644285 | Qv33364 | AA1 |
| D3 | HQ644288 | Rw649 | AA1 |
| D3 | HQ644289 | Rw677 | AA1 |
| D3 | HQ644265 | Qv04917 | AA1 |
| D3 | HQ644255 | Qv00346 | AA1 |
| D3 | HQ644269 | Qv13040 | AA1 |
| D3 | HQ644253 | Qv00079 | AA1 |
| D3 | HQ644278 | Qv23856 | AA1 |
| D3 | HQ644276 | Qv21730 | AA1 |

**Supplementary data 2:** Balanced validation matrices for random-forest models to assess HPV16-lineage. The same number of samples for each lineage (n = 50) has been randomly selected according to the Maximum Likelihood Tree (MLT) classification. Lineage has been assigned with the RF model and compared with the previous classification. The procedure has been repeated 4 times with different sets of samples.


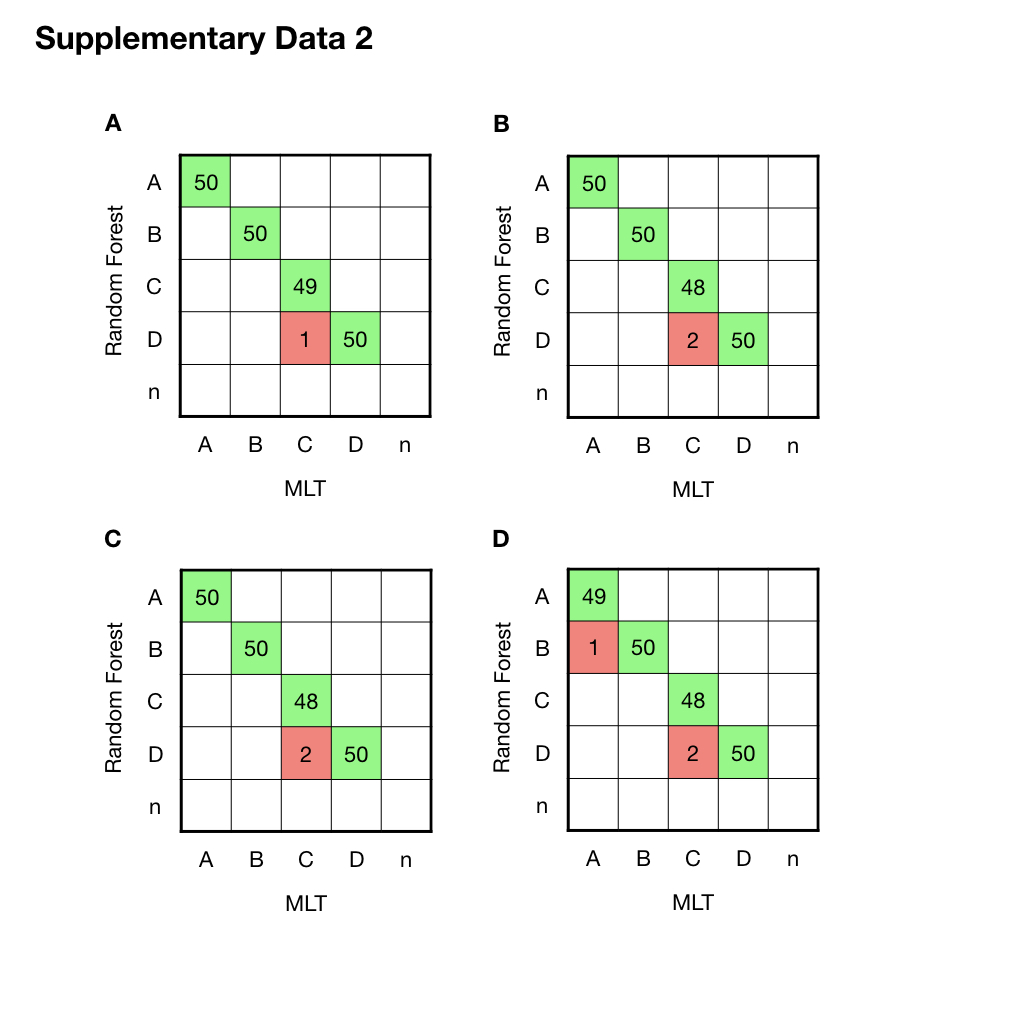

Supplement: Supplementary file 1 [file Table_1.docx]
